# Micropropagation of *Philodendron* ‘White Knight’ via Shoot Regeneration from Petiole Explants

**DOI:** 10.3390/plants14111714

**Published:** 2025-06-04

**Authors:** Iro Kang, Iyyakkannu Sivanesan

**Affiliations:** 1 Department of Horticulture, College of Agriculture & Natural Resources, Michigan State University, East Lansing, MI 48824, USA; kanghye4@msu.edu; 2 Department of Environmental Health Science, Institute of Natural Science and Agriculture, Konkuk University, 1 Hwayang-dong, Gwangjin-gu, Seoul 05029, Republic of Korea

**Keywords:** adventitious shoot, auxin, cytokinin, in vitro propagation, silver nanoparticles

## Abstract

*Philodendron* ‘White Knight’ is a popular climbing evergreen plant typically propagated through stem cuttings. However, this method is slow and inefficient, making it challenging to meet the rising market demand. In vitro propagation could enhance the multiplication of this cultivar. However, research on its in vitro propagation is limited. Therefore, the objective of the present study was to establish an efficient micropropagation technique to mass-produce *Philodendron* ‘White Knight’ to meet the market demand. We investigate the impact of silver nanoparticles (Ag NPs) on the surface sterilization of *Philodendron* ‘White Knight’ petioles, the role of plant growth regulators in adventitious shoot regeneration and shoot multiplication, and the effect of auxins on the rooting ability of *Philodendron* ‘White Knight’ microshoots. There are few stages in plant micropropagation. The establishment of aseptic culture is the first and most important stage. For *Philodendron* ‘White Knight’, aseptic petiole explants (100%) were obtained after treatment with 40 mg L^−1^ Ag NPs for 60 min. This was followed by adventitious shoot induction, and the highest rate of adventitious shoot induction (52.6%) and the maximum shoot number (13.9 shoots per petiole) were achieved on Murashige and Skoog shoot multiplication B (MS-B) medium with 20 µM of 2-isopentenyl adenine (2-IP) and 5.0 µM of naphthalene acetic acid (NAA). The shoot multiplication stage was achieved with the highest number of shoots (34 shoots per shoot tip) with a length of 5.1 cm, which was obtained on MS-B medium with 5.0 µM 2-IP and 2.5 µM NAA. All the microshoots produced roots during the root induction stage with the maximum root number (8.2 roots per shoot), and the greatest plantlet height (9.1 cm) was achieved on half-strength Murashige and Skoog medium containing indole-3-butyric acid (10.0 μM). The rooted plantlets of *Philodendron* ‘White Knight’ were transplanted into a substrate composed of 10% peat moss, 50% orchid stone, and 40% coconut husk chips and acclimatized in a greenhouse environment, achieving a survival rate of 100%. This micropropagation protocol can be used for the commercial production of *Philodendron* ‘White Knight’.

## 1. Introduction

The genus *Philodendron* Schott, belonging to the Araceae family, comprises 625 species native to South America, Central America, the Caribbean, and parts of Colombia, French Guiana, and Guyana [1]. Many *Philodendron* species are popular indoor plants due to their attractive foliage and low maintenance requirements, with several cultivars (cv.) emerging in recent decades [2]. *Philodendron* cultivars with orange, red, and yellow foliage, either variegated or non-variegated, tend to attract more attention and command higher market value, especially the variegated varieties [2,3]. Although they are typically propagated through stem cuttings and seeds, these methods do not meet market demand due to the lack of healthy plant materials and the lengthy propagation process [2,4,5]. In contrast, in vitro propagation techniques offer excellent alternatives for mass-propagating *Philodendron* species and cultivars, utilizing less plant material and enabling year-round production with reduced space and a shorter time to meet market demand [2].

Micropropagation is a powerful in vitro plant culture technique widely used for the commercial propagation of various ornamental plants [6], including *Philodendron billietiae* Croat [7], *Philodendron bipinnatifidum* Schott ex Endl. [8], *Philodendron erubescens* cv. Pink Princess [5], *Philodendron pertusum* Kunth & C.D.Bouché [9], *Philodendron selloum* Koch [10], *Philodendron tuxtlanum* G.S.Bunting [11], *Philodendron* cv. Birkin [12], Imperial Green, Imperial Red, and Imperial Rainbow [2], as well as Blue Mistic, Painted Lady, Pink Prince, Pluto, Royal Queen, and Green Emperor [4]. The authors have utilized various explants, including axillary buds, axillary shoots, leaf laminae, nodes, petioles, shoot tips, and stem nodal segments, cultured on media containing a range of plant growth regulators, such as 2,4-dichlorophenoxyacetic acid (2,4-D), 2-isopentenyl adenine (2IP), benzyladenine (BA)/6-benzylaminopurine (BAP), indole-3-butyric acid (IBA), kinetin (KN), naphthalene acetic acid (NAA), and thidiazuron (TDZ), either alone or in combination, to achieve adventitious shoot regeneration or axillary shoot multiplication. However, shoot induction depends on explant type, culture medium composition, genotype, and plant growth regulators [2]. Therefore, the development of a micropropagation protocol for the mass-production of a new cultivar of *Philodendron* is necessary.

Micropropagation involves multiple stages, including the establishment of aseptic culture, shoot multiplication, shoot elongation, rooting, and acclimatization [6]. Although all stages are equally important, the initial establishment of in vitro aseptic culture is a critical stage, and contamination poses a significant challenge. Success depends on effectively disinfecting explant surfaces while preserving their viability [13]. *Philodendron* explants were often surface disinfected with 70–95% ethyl alcohol (EtOH), 0.16–5.0% sodium hypochlorite (NaOCl), and 0.05–0.1% mercuric chloride (HgCl_2_) [4,6,9,12,14,15]. Nanoparticles (NPs) effectively combat various bacteria, fungi, and viruses that can contaminate plant explants [16]. Their small size allows them to penetrate microbial cell walls easily, making them more efficient than traditional disinfectants. NPs can be utilized in lower concentrations, minimizing toxicity risks to plant tissues while remaining effective. Furthermore, they are generally more environmentally friendly than HgCl_2_, making them a promising alternative for disinfecting plant tissue culture explants [17,18]. Silver (Ag) NPs are environmentally friendly chemicals that have been used to eliminate contaminants in plant tissue culture [15] because of their powerful antimicrobial properties. Recently, Lara-Ascencio et al. [18] demonstrated that the Ag NPs treatment reduces the presence of contaminants in the *Philodendron xanadu* Croat explants. *Philodendron *‘White Knight’ is a climbing evergreen plant native to South America. It is one of the popular *Philodendron* cultivars due to its variegated foliage and is used as both an indoor and garden plant. It is commonly multiplied through stem cutting; however, vegetative propagation is slow and requires effective alternative methods for multiplying this important cultivar to meet market demand. So far, no reports have documented the in vitro propagation of *Philodendron* ‘White Knight’. Thus, this study aimed to develop an efficient micropropagation protocol for the mass-production of *Philodendron* ‘White Knight’ through adventitious shoot regeneration using petiole explants.

## 2. Materials and Methods

### 2.1. Selection of Plant Materials and Establishment of Aseptic Explants

Petioles were collected from fully developed leaves of three-year-old *Philodendron* ‘White Knight’ plants cultivated in a polyhouse. The cut ends of the petioles were sealed with an instant adhesive, thoroughly cleaned under running tap water for 20 min, and then washed with distilled water (DH_2_O) containing 0.15% (*w*/*v*) polyvinylpyrrolidone (PVP, Sigma-Aldrich, Inc., St. Louis, MO, USA). Petioles were surface disinfected in laminar airflow by treating them with 70% (*v*/*v*) EtOH (95%, DaeJung Chemicals & Metals, Siheung-si, Republic of Korea) for 30 s; rinsed three times in sterile DH_2_O containing 0.15% PVP (SDH_2_O-PVP) and immersed in 1.0% (*v*/*v*) NaOCl solution for 12 min; and washed five times with SDH_2_O-PVP. To study the effect of Ag NPs (<100 nm particle size, 99.5% purity, Sigma-Aldrich, Inc., St. Louis, MO, USA) on surface disinfection, the NaOCl-treated petioles were immersed in 10, 20, 40, or 80 mg L^−1^ Ag NPs for 30, 60, or 90 min and then washed five times with SDH_2_O-PVP. The sealed ends were discarded, and the petioles were cut transversely into 0.4–0.6 mm segments and then cultured on Murashige and Skoog shoot multiplication B (MS-B) medium (Duchefa Biochemie, Haarlem, The Netherlands) containing 8.0 µM BA. All media contained 0.6 g L^−1^ activated charcoal (AC), 30 g L^−1^ sucrose, 0.8 g L^−1^ agar (Plant Agar, Duchefa Biochemie, Haarlem, The Netherlands), and the pH of each medium was adjusted to 5.70–5.80 before autoclaving at 123 °C for 23 min. Five petioles of *Philodendron* ‘White Knight’ were inoculated in each culture bottle, with nine culture bottles assigned to each treatment. The factorial experiment was conducted twice using a randomized block design. The cultures were kept at 23 ± 1 °C under a 12 h photoperiod provided by white light-emitting diodes (WLEDs) with a photosynthetic photon flux density (PPFD) of 18–23 μMol s^−1^ m^−2^. The rates of contamination and survival of *Philodendron* ‘White Knight’ explants were recorded after 21 days of incubation. Explants that turned black were deemed dead, while those that remained purple or increased in size were considered alive.

### 2.2. Induction of Adventitious Shoots

To study the impact of PGRs on adventitious shoot induction, the explants were prepared from petioles treated with 40 mg L^−1^ Ag NPs for 60 min and cultured on MS-B medium containing 5, 10, 20, or 30 µM of BA or 2-IP combined with 2.5 or 5.0 µM of NAA or IBA. The medium MS-B, devoid of plant growth regulators (PGRs), served as a control. All PGRs were procured from Duchefa Biochemie, Haarlem, The Netherlands. Five petioles of *Philodendron* ‘White Knight’ were inoculated in each culture bottle, with twelve culture bottles assigned to each treatment. The factorial experiment was conducted twice using a randomized block design. The cultures were kept at 23 ± 1 °C under a 12 h photoperiod provided by WLEDs with a PPFD of 35–40 μMol s^−1^ m^−2^. The number of shoots per *Philodendron* ‘White Knight’ petiole was recorded after 13 weeks of incubation.

### 2.3. Multiplication of Micro-Shoots

The adventitious shoot buds were transferred to PGR-free MS-B medium and subcultured at four-week intervals. After three subcultures, the in vitro developed shoots were utilized to obtain shoot tip explants. For shoot multiplication, shoot tips were cultured on the MS-B medium containing 2.5, 5.0, or 10.0 µM 2-IP combined with 2.5 µM NAA. The medium MS-B, devoid of 2-IP and NAA, served as a control. Six shoot tips of *Philodendron* ‘White Knight’ were inoculated in each culture bottle, with 15 culture bottles assigned to each treatment. The factorial experiment was conducted twice using a randomized block design. The cultures were kept at 23 ± 1 °C under a 14 h photoperiod provided by WLEDs with a PPFD of 45–50 μMol s^−1^ m^−2^. The number of shoots per *Philodendron* ‘White Knight’ shoot bud and shoot length (mm) were recorded after 13 weeks of incubation.

### 2.4. Root Induction of Micro-Shoots

Well-developed shoots separated from the shoot clusters were transferred to half-strength Murashige and Skoog (MS) [19] medium containing 0, 2.5, 5.0, 10.0, or 20.0 µM NAA or IBA to induce roots. Six shoots of *Philodendron* ‘White Knight’ were inoculated in each culture bottle, with 15 culture bottles assigned to each treatment. The factorial experiment was conducted twice using a randomized block design. The cultures were kept at 23 ± 1 °C under a 14 h photoperiod provided by WLEDs with a PPFD of 60–65 μMol s^−1^ m^−2^. The number of roots per *Philodendron* ‘White Knight’ shoot and plantlet height were recorded after 8 weeks of incubation.

### 2.5. Acclimatization

The well-developed 300 *Philodendron* ‘White Knight’ plantlets were separated from the rooting medium, thoroughly washed to remove any traces of rooting medium, and then transplanted into trays containing a mixture of peat moss (10%), orchid stone (50%), and coconut husk chips (40%). They were subsequently acclimatized in a greenhouse at a temperature of 20–25 °C and a relative humidity of 95–100% for 2 weeks, which was gradually reduced to 60%. The plants were fertigated with ¼ MS nutrients, and the survival of the plantlets was recorded after 6 weeks of cultivation.

### 2.6. Statistical Analysis

The in vitro experiments were conducted in a randomized design with three replications. The data were subjected to analysis of variance (ANOVA) tests, and means were distinguished by Duncan’s multiple range test (DMRT) at *p* ≤ 0.05. The percent values were transformed using arcsine square root (√*p*) to normalize the error distribution before ANOVA. The statistical analyses were performed using SAS Release 9.4.

## 3. Results and Discussion

### 3.1. Impact of Ag NPs on the Surface Sterilization of Philodendron ‘White Knight’ Petiole Explants

The petiole explants of *Philodendron* ‘White Knight’ treated with EtOH and NaOCl (control) yielded 25.9% aseptic explants, while 74.1% of the petioles were contaminated with bacteria and fungi; however, the survival rate of *Philodendron* ‘White Knight’ explants remained unaffected. The percentage of aseptic cultures increased when the petioles were treated with Ag NPs compared to the control (Table 1). The positive effect of Ag NPs on the surface disinfection of explants has also been documented in *Ardisia mamillata* [12], *Begonia tuberous* [20], *Limonium sinuatum* ‘White’ [21], *Monstera deliciosa* ‘Thai Constellation’ [22], *Ocimum basilicum* ‘Italian Large Leaf’, *Salvia farinecae*, and *Thymus vulgaris* [23]. Increasing the concentration of Ag NPs and soaking period reduced explant contamination; however, higher concentrations and a longer soaking period also resulted in a decrease in the explant survival rate (Table 1). Similar results have been observed in *A. mamillata* [13], *Kaempferia parviflora* [24], *M. deliciosa* ‘Thai Constellation’ [22], *O. basilicum* ‘Italian Large Leaf’, S. *farinecae*, and *T. vulgaris* [23]. In this study, soaking *Philodendron *‘White Knight’ petiole explants in 10 mg L^−1^ silver nanoparticles for 30, 60, and 90 min resulted in 29.3%, 40.1%, and 51.4% of aseptic explants, respectively. Additionally, the survival rate (100%) of explants remained unaffected. Among the various Ag NPs treatments, 40 mg L^−1^ Ag NPs for 60 min was found to be the most effective, resulting in 100% aseptic explants with a 100% survival rate. In contrast, the survival rate of explants decreased when treated with 40 mg L^−1^ Ag NPs for 90 min (Table 1).

### 3.2. Effects of PGRs on Adventitious Shoot Regeneration from Petiole Explants of Philodendron ‘White Knight’

#### 3.2.1. Combination of 2-IP and Auxins (NAA and IBA)

**Figure 1:**
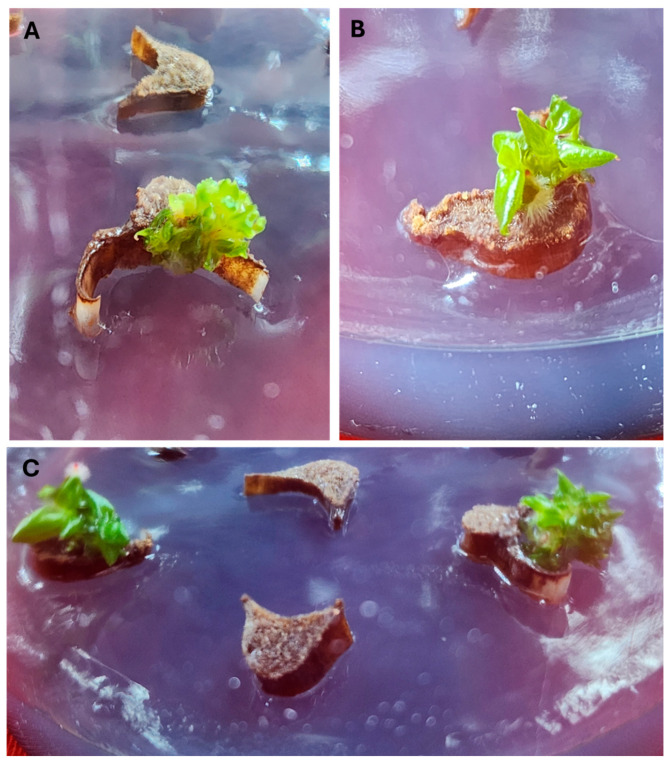
Adventitious shoot regeneration. Shoots developed from *Philodendron* ‘White Knight’ petioles cultured on MS-B medium with 2-IP and NAA (**A**), 2-IP and IBA (**B**), and BA and IBA (**C**).

Direct adventitious shoot regeneration is an effective technique that enhances efficiency and sustainability in commercial plant propagation [25] and is essential for *Agrobacterium*-mediated genetic transformation [26]. It has been reported that the petiole segments of *Philodendron scandens* [27] and *Philodendron* ‘Imperial Green’ [2] do not produce shoots. In this study, the petiole explants of *Philodendron* ‘White Knight’ did not yield adventitious shoots on the MS-B medium lacking PGRs or containing 5.0 µM 2-IP plus auxins, and they died after 13 weeks of culture. However, adventitious shoot buds developed from the explants within 8 weeks of culture when the MS-B medium was fortified with higher concentrations of 2-IP and auxins (Figure 1A,B). It has been revealed that supplementing both auxin and cytokinin can induce adventitious shoot regeneration in several members of the Araceae family, including *Aglonema* ‘Lady Valentine’ [28], *Anthurium andraeanum* [29], *Epipremnum aureum* [30], *Spathiphyllum wallsii* ‘Domino’ [31], and *Zamioculcas zamiifolia* [32]. When the MS-B medium was fortified with 10–30 µM of 2-IP and 2.5 µM of NAA, 15–38.9% of *Philodendron* ‘White Knight’ petioles developed 2.8–7.0 adventitious shoots per explant (Figure 1A). The highest adventitious shoot induction (52.6%) and the maximum shoot production (13.9 shoots per explant) were achieved on MS-B medium with 20 µM of 2-IP and 5.0 µM of NAA (Table 2). This result surpassed previous findings, in which only 2.8% of petioles from *Philodendron* Imperial Red and 11.1% from Imperial Rainbow produced shoots [2]. When 2-IP was combined with IBA, 14.5–34.9% of *Philodendron* ‘White Knight’ petioles developed 1.9–6.3 adventitious shoots per explant (Table 2). Of the 2-IP and IBA combinations studied, 20 µM of 2-IP and 5.0 µM of IBA were found to be the best for shoot production (34.9% of explants induced 6.3 shoots per petiole).

#### 3.2.2. Combination of BA and Auxins (NAA and IBA)

When BA (10–30 µM) was combined with NAA (2.5 or 5.0 µM), 8.3–38.8% of petioles produced 1.2–4.8 shoots per explant. The maximum adventitious shoot induction (38.8%) and the highest number of shoots (4.8 shoots per petiole) were achieved on MS-B medium containing 20 µM BA and 2.5 µM NAA. Increasing concentrations of both BA and NAA reduced the shoot production potential of *Philodendron* ‘White Knight’ petiole explants. However, the shoot regeneration ability did not differ significantly when compared to MS-B medium supplemented with 30 μM BA + 5 μM IBA (Table 3). The presence of BA and IBA in the MS-B medium enhanced shoot production, with the best adventitious shoot induction at 46.1% and the maximum number of shoots at 7.1 shoots per petiole achieved on MS-B medium with 20 µM BA and 5.0 µM IBA (Table 3, Figure 1C). The results (Table 2 and Table 3) indicated that adventitious shoot production from petiole explants of *Philodendron* ‘White Knight’ was influenced by the type of cytokinin and auxin. The petioles of *Philodendron* ‘White Knight’ showed the best response (52.6% of explants induced 13.9 shoots per petiole) to MS-B medium containing 2-IP (20 µM) and NAA (5 µM). The presence of 2-IP and NAA in the culture medium has been shown to effectively promote shoot formation from petioles of *Bixa orellana* [33].

### 3.3. Shoot Multiplication

**Figure 2:**
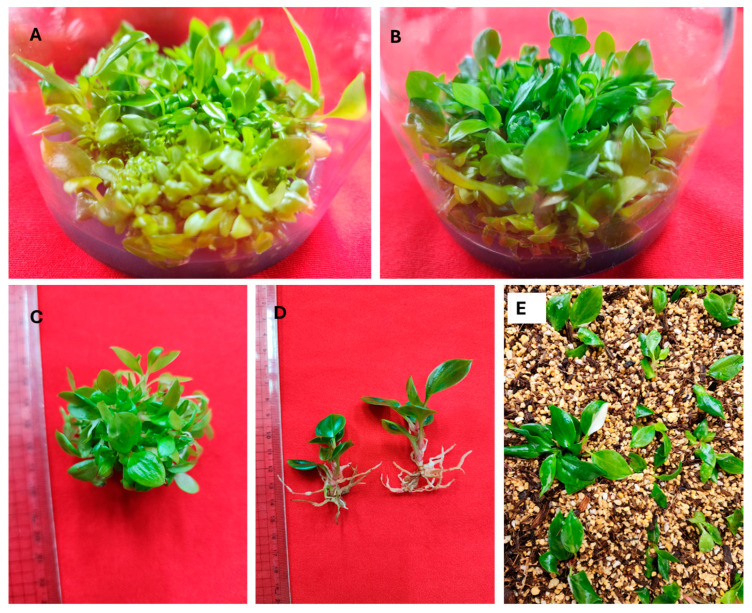
Shoot multiplication, root induction of micro-shoots, and plantlet acclimatization. Multiple shoot formation from shoot tips of *Philodendron* ‘White Knight’ on MS-B medium containing 5.0 µM 2-IP and 2.5 µM NAA after 6 weeks (**A**) and 13 weeks (**B**) of cultivation; (**C**) multiple shoot cluster; (**D**) micro-shoots of *Philodendron* ‘White Knight’ rooting in ½ MS medium with 10.0 µM IBA; (**E**) acclimatized *Philodendron* ‘White Knight’ plantlets.

Chen et al. [2] utilized in vitro regenerated shoots of *Philodendron* cultivars as the explant (node) source to examine the impact of cytokinins (BA, KN, and TDZ) on shoot multiplication. Similarly, the shoot multiplication of *Philodendron* cultivars or species has been achieved using in vitro-derived nodes, protocorm-like bodies, and shoot tips on culture media containing BA alone or in combination with auxins [5,7,8,34]. These studies have demonstrated that BA/BAP, whether used alone or in combination with auxins, is more effective for shoot multiplication than other cytokinin treatments. In this study, the results indicated that a combination of 2-IP and NAA produced a higher number of shoots. Therefore, the impact of various concentrations of 2-IP combined with 2.5 µM NAA was examined on multiple shoot induction from in vitro-derived shoot tips of *Philodendron* ‘White Knight’. The shoot tips of *Philodendron* ‘White Knight’ cultured on MS-B medium devoid of 2-IP and NAA produced multiple shoots (4.9 shoots per shoot tip) with a length of 6.4 cm (Table 4). In contrast, Alawaadh et al. [8] reported that shoot tip explants of *Philodendron bipinnatifidum* developed only a single shoot on PGR-free media. On the other hand, *P. erubescens *‘Pink Princess’ protocorm-like bodies developed 4.4 shoots on PGR-free medium [5]. The addition of 2-IP and NAA to MS-B medium significantly increased the number of shoots per *Philodendron* ‘White Knight’ shoot tip compared to the control, while the length of the shoot significantly decreased in a dose-dependent manner. The highest number of shoots (34 shoots per shoot tip) with a length of 5.1 cm was obtained on MS-B medium containing 5.0 µM 2-IP and 2.5 µM NAA (Table 4, Figure 2A–C). Likewise, shoot multiplication has been achieved in the presence of 2-IP and NAA in *Allium sativum* [35], *Philodendron bipinnatifidum* [8], and *Philodendron goeldii* [36].

### 3.4. Rooting of Micro-Shoots

**Figure 3:**
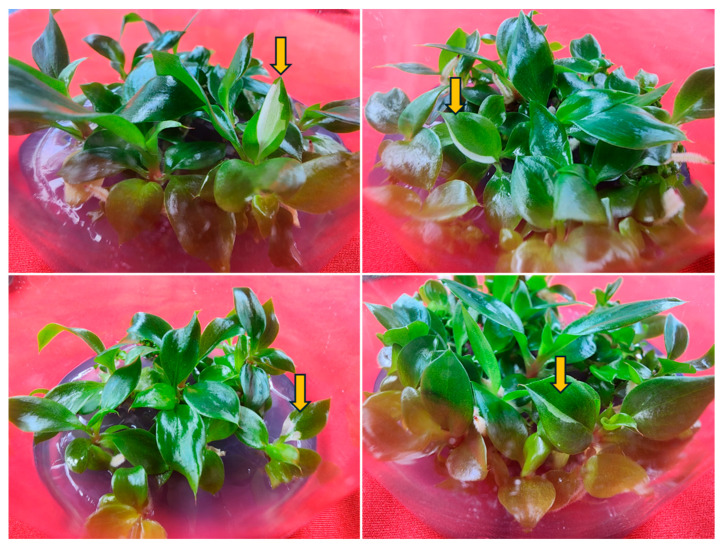
In vitro regenerated normal and variegated (arrow) plantlets of *Philodendron* ‘White Knight’.

Rooting is a crucial stage of micropropagation influenced by the composition of the shoot induction medium, the rooting medium composition, the type and concentration of auxins, as well as the genotype and plant species [6]. In vitro rooting of *Philodendron* cultivars and species has been achieved on basal medium without [4,10] or with auxins [2,5,7,8,9,14]. Previous studies have shown that in vitro rooting of *Philodendron* cultivars and species is best achieved on a medium containing IBA or NAA [2,5,7,8,9,14]. Thus, this study investigated the influence of IBA and NAA on the rooting of *Philodendron* ‘White Knight’ shoots. In the control medium (auxin-free ½ MS), 18.9% of shoots successfully developed an average of 2.2 roots, with an average plantlet height of 3.9 cm (Table 5). The inclusion of IBA or NAA at concentrations ranging from 2.5 to 20.0 µM to the ½ MS medium significantly increased the rooting percentage (52.2–100%), the average number of roots (2.7–8.2), and the plantlet height (4.3–5.8) compared to the control. However, it is noteworthy that elevated concentrations of NAA, particularly at 20.0 µM, resulted in a marked decrease in plantlet height, which measured only 3.1 cm compared to the control. Of the various concentrations (2.5–20.0 μM) of auxins tested, 10.0 μM IBA resulted in the highest root induction (100%), the maximum root number (8.2 roots per shoot), and the greatest plantlet height (9.1 cm) (Table 5). Among the two auxins tested, IBA was found to be the best for in vitro rooting of *Philodendron* ‘White Knight’ shoots (Figure 2D). The beneficial effects of IBA on the rooting of Araceae members have been shown in *Aglaonema* ‘Lady Valentine’ [37], *M. deliciosa* ‘Thai Constellation’ [22], *Philodendron* cultivars [2], *Philodendron* ‘Birkin’ [12], *P. erubescens* ‘Pink Princess’ [5], *P. pertusum* [9], *Philodendron xanadu* [18], and *Spathiphyllum wallsii* ‘Domino’ [31]. In this study, most shoots that regenerated in vitro during the rooting phase displayed normal green leaves; however, a subset of shoots exhibited variegated leaves (Figure 3).

### 3.5. Acclimatization

Acclimatization is the final stage of micropropagation, influenced mainly by the growth substrate. *Philodendron* cultivars and species have been successfully acclimatized using a mixture of various substrates such as peat moss and perlite [2,8], river sand, farmyard manure, and soil [4], perlite, vermiculite, and peat moss cocopeat [5], sand, soil, and farmyard manure [9], and coir and *Luffa* sponge [14]. In this study, both the normal and variegated plantlets of *Philodendron* ‘White Knight’ were successfully transplanted into a substrate composed of 10% peat moss, 50% orchid stone, and 40% coconut husk chips and acclimatized (Figure 2E) in a greenhouse environment, achieving a survival rate of 100%.

## 4. Conclusions

An efficient micropropagation protocol was successfully established for the commercial propagation of *Philodendron* ‘White Knight’. This micropropagation protocol consisted of four stages: the establishment of aseptic petiole explants, induction of adventitious shoot formation, microshoot multiplication, rooting of the micro-shoots, and acclimatization, which can be utilized in breeding programs aimed at enhancing this cultivar.

## Figures and Tables

**Table 1:** Effects of Ag NPs concentration and soaking period on the surface disinfection of *Philodendron *‘White Knight’ Petiole Explants.

Ag NPs (mg L^−1^)	Soaking Period (min)	Aseptic Explant (%)	Survival (%)
Control	0	25.9 ± 1.6 h	100 ± 0.0 a
10	30	29.3 ± 1.2 h	100 ± 0.0 a
20	30	34.8 ± 1.2 g	100 ± 0.0 a
40	30	47.8 ± 1.3 e	100 ± 0.0 a
80	30	54.7 ± 1.7 d	100 ± 0.0 a
10	60	40.1 ± 1.4 f	100 ± 0.0 a
20	60	72.1 ± 2.1 c	100 ± 0.0 a
40	60	100 ± 0.0 a	100 ± 0.0 a
80	60	100 ± 0.0 a	77.0 ± 3.0 d
10	90	51.4 ± 1.5 d	100 ± 0.0 a
20	90	81.2 ± 1.9 b	92.7 ± 1.5 b
40	90	100 ± 0.0 a	84.9 ± 2.4 c
80	90	100 ± 0.0 a	68.2 ± 2.3 e

Means ± standard errors in a column that share the same letter(s) are not significantly different, as determined by DMRT at *p* ≤ 0.05.

**Table 2:** Effect of different concentrations of 2-IP combined with NAA and IBA on adventitious shoot regeneration from petiole explants of *Philodendron *‘White Knight’.

PGRs (µM)			Shoot Induction (%)	Number of Shoots/Explant
2-IP	NAA	IBA		
0	0.0	0.0	0.0 ± 0.0 i	0.0 ± 0.0 h
5	2.5	0.0	0.0 ± 0.0 i	0.0 ± 0.0 h
10	2.5	0.0	15.0 ± 1.9 h	2.8 ± 0.4 fg
20	2.5	0.0	38.9 ± 1.7 bc	7.0 ± 0.7 bc
30	2.5	0.0	33.0 ± 1.4 de	4.7 ± 0.3 de
5	5.0	0.0	0.0 ± 0.0 i	0.0 ± 0.0 h
10	5.0	0.0	30.0 ± 2.2 ef	4.2 ± 0.7 def
20	5.0	0.0	52.6 ± 2.0 a	13.9 ± 1.0 a
30	5.0	0.0	42.2 ± 2.5 b	8.4 ± 1.0 b
5	0.0	2.5	0.0 ± 0.0 i	0.0 ± 0.0 h
10	0.0	2.5	14.5 ± 1.3 h	1.9 ± 0.4 g
20	0.0	2.5	21.2 ± 1.9 g	5.4 ± 0.7 cd
30	0.0	2.5	17.8 ± 1.8 gh	3.5 ± 0.6 efg
5	0.0	5.0	0.0 ± 0.0 i	0.0 ± 0.0 h
10	0.0	5.0	20.6 ± 1.2 g	2.2 ± 0.4 g
20	0.0	5.0	34.9 ± 2.0 cd	6.3 ± 0.6 c
30	0.0	5.0	28.2 ± 2.1 f	3.4 ± 0.5 efg

Means ± standard errors in a column that share the same letter(s) are not significantly different, as determined by DMRT at *p* ≤ 0.05.

**Table 3:** Effect of different concentrations of BA combined with NAA and IBA on adventitious shoot regeneration from petiole explants of *Philodendron *‘White Knight’.

PGRs (µM)			Shoot Induction (%)	Number of Shoots/Explant
BA	NAA	IBA		
0	0.0	0.0	0.0 ± 0.0 i	0.0 ± 0.0 g
5	2.5	0.0	0.0 ± 0.0 i	0.0 ± 0.0 g
10	2.5	0.0	22.8 ± 1.9 d	2.2 ± 0.3 de
20	2.5	0.0	38.8 ± 2.0 b	4.8 ± 0.4 b
30	2.5	0.0	30.9 ± 1.2 c	2.9 ± 0.4 cd
5	5.0	0.0	0.0 ± 0.0 i	0.0 ± 0.0 g
10	5.0	0.0	10.8 ± 1.3 gh	1.6 ± 0.2 ef
20	5.0	0.0	16.7 ± 1.7 ef	3.0 ± 0.4 cd
30	5.0	0.0	8.3 ± 1.2 h	1.2 ± 0.2 f
5	0.0	2.5	0.0 ± 0.0 i	0.0 ± 0.0 g
10	0.0	2.5	18.9 ± 1.4 e	1.8 ± 0.2 ef
20	0.0	2.5	24.8 ± 1.6 d	2.7 ± 0.2 cd
30	0.0	2.5	13.9 ± 1.4 fg	1.4 ± 0.2 ef
5	0.0	5.0	0.0 ± 0.0 i	0.0 ± 0.0 g
10	0.0	5.0	32.2 ± 2.2 c	3.3 ± 0.4 c
20	0.0	5.0	46.1 ± 1.8 a	7.1 ± 0.6 a
30	0.0	5.0	39.3 ± 1.5 b	4.6 ± 0.4 b

Means ± standard errors in a column that share the same letter(s) are not significantly different, as determined by DMRT at *p* ≤ 0.05.

**Table 4:** Effect of concentrations of 2-IP along with NAA on shoot multiplication.

2-IP (µM)	NAA (µM)	Number of Shoots/Explant	Shoot Length (cm)
0.0	0.0	4.9 ± 0.7 d	6.4 ± 0.7 a
2.5	2.5	11.2 ± 1.0 c	5.1 ± 0.7 ab
5.0	2.5	34.0 ± 2.4 a	3.7 ± 0.6 bc
10.0	2.5	21.4 ± 1.5 b	2.9 ± 0.5 c

Means ± standard errors in a column that share the same letter(s) are not significantly different, as determined by DMRT at *p* ≤ 0.05.

**Table 5:** Effect of Auxins on Rooting of *Philodendron *‘*White Knight*’ Shoots.

Auxin (µM)		Rooting (%)	Number of Roots/Explant	Plantlet Height (cm)
IBA	NAA			
0.0	0.0	18.9 ± 2.0 f	2.2 ± 0.1 d	3.9 ± 0.3 ef
2.5	0.0	52.2 ± 2.1 e	3.6 ± 0.4 cd	5.8 ± 0.2 cd
5.0	0.0	75.1 ± 3.1 c	4.7 ± 0.4 bc	7.3 ± 0.4 b
10.0	0.0	100 ± 0.0 a	8.2 ± 0.7 a	9.1 ± 0.3 a
20.0	0.0	87.2 ± 2.6 b	3.1 ± 0.3 d	6.4 ± 0.3 c
0.0	2.5	55.8 ± 3.2 e	2.7 ± 0.4 d	5.3 ± 0.5 d
0.0	5.0	91.7 ± 1.7 b	5.2 ± 0.3 b	4.3 ± 0.2 e
0.0	10.0	74.4 ± 1.8 c	3.2 ± 0.5 d	3.6 ± 0.2 ef
0.0	20.0	65.9 ± 2.1 d	2.9 ± 0.4 d	3.1 ± 0.2 f

Means ± standard errors (SE) in a column that share the same letter(s) are not significantly different, as determined by DMRT at *p* ≤ 0.05.

## Data Availability

Data are contained within the article.

## Abbreviations

The following abbreviations are used in this manuscript:

Ag NPs	silver nanoparticles
MS-B	Murashige and Skoog shoot multiplication B
2-IP	2-isopentenyl adenine
IBA	indole-3-butyric acid
NAA	naphthalene acetic acid
BA	benzyladenine
PPFD	photosynthetic photon flux density
